# Cytotoxicity and antibacterial susceptibility assessment of a newly developed pectin–chitosan polyelectrolyte composite for dental implants

**DOI:** 10.1038/s41598-024-68020-7

**Published:** 2024-07-23

**Authors:** Mohammed Hussein M. Alsharbaty, Ghassan A. Naji, Ban A. Ghani, Michael Schagerl, Maha A. Khalil, Sameh S. Ali

**Affiliations:** 1 Branch of Prosthodontics, College of Dentistry, University of Al-Ameed, Karbala, Iraq; 2 College of Dentistry, AL-Iraqia University, Baghdad, Iraq; 3 Department of Oral Diagnostic Sciences, College of Dentistry, University of Baghdad, Baghdad, Iraq; 4 Department of Functional and Evolutionary Ecology, University of Vienna, Djerassiplatz 1, 1030 Vienna, Austria; 5 Biology Department, College of Science, Taif University, 21944 Taif, Saudi Arabia; 6 Botany Department, Faculty of Science, Tanta University, Tanta, 31527 Egypt

**Keywords:** Pectin, Chitosan, Titanium, Antibacterial susceptibility, Cytotoxicity, Dental implants, Biotechnology, Drug discovery, Medical research

## Abstract

Biopolymers such as chitosan and pectin are currently attracting significant attention because of their unique properties, which are valuable in the food industry and pharmaceutical applications. These properties include non-toxicity, compatibility with biological systems, natural decomposition ability, and structural adaptability. The objective of this study was to assess the performance of two different ratios of pectin–chitosan polyelectrolyte composite (PCPC) after applying them as a coating to commercially pure titanium (CpTi) substrates using electrospraying. The PCPC was studied in ratios of 1:2 and 1:3, while the control group consisted of CpTi substrates without any coating. The pull-off adhesion strength, cytotoxicity, and antibacterial susceptibility tests were utilized to evaluate the PCPC coatings. In order to determine whether the composite coating was the result of physical blending or chemical bonding, the topographic surface parameters were studied using Fourier transform infrared spectroscopy (FTIR) and atomic force microscopy (AFM). PCPC (1:3) had the highest average cell viability of 93.42, 89.88, and 86.85% after 24, 48, and 72 h, respectively, as determined by the cytotoxicity assay, when compared to the other groups. According to the Kirby–Bauer disk diffusion method for testing antibacterial susceptibility, PCPC (1:3) showed the highest average diameter of the zone of inhibition, measuring 14.88, 14.43, and 11.03 mm after 24, 48, and 72 h of incubation, respectively. This difference was highly significant compared to Group 3 at all three time periods. PCPC (1:3) exhibited a significantly higher mean pull-off adhesion strength (521.6 psi) compared to PCPC (1:2), which revealed 419.5 psi. PCPC (1:3) coated substrates exhibited better surface roughness parameters compared to other groups based on the findings of the AFM. The FTIR measurement indicated that both PCPC groups exhibited a purely physical blending in the composite coating. Based on the extent of these successful in vitro experiments, PCPC (1:3) demonstrates its potential as an effective coating layer. Therefore, the findings of this study pave the way for using newly developed PCPC after electrospraying coating on CpTi for dental implants.

## Introduction

The utilization of dental implants has been widespread in the fabrication of fixed, detachable, and maxillofacial prostheses, demonstrating a highly promising rate of success^1^. Titanium or titanium alloys are often regarded as the primary choice for implant materials because of their biocompatibility, exceptional mechanical qualities, and resistance to corrosion^2^. The process of titanium directly attaching with bone is referred to as osseointegration^3^. Titanium and titanium alloys are widely regarded as the optimal and preferred materials for dental implants due to their outstanding biocompatibility and ability to integrate with bone tissue^4^. Commercially pure titanium (CpTi) has excellent corrosion resistance, exceptional biocompatibility, spontaneous production of oxide layers, high specific strength, and is non-toxic^4^. However, there is still need for improvement, namely in terms of stabilizing the implant and enhancing its integration at the bone-biomaterial interface^5^.

The leading cause of implant failure is insufficient bone formation and attachment to the implant surface^6^. The significance of tissue interface and implant surface macro and micro-characteristics in osseointegration has been widely documented^7^. Diverse inorganic and organic nanocoating substances have been the subject of research in an effort to develop materials for dental implant coatings that provide optimal benefits^8^. Polysaccharides, such as glycosaminoglycans, have been employed in the coating of implant surfaces^9^. In contrast to proteins, which function as nanocoating materials, polysaccharides maintain their cell-attracting properties at the coated surface for an extended duration^10^. Polysaccharides can be obtained from sources originating from plants, animals, and humans^11,12^.

Chitosan is a polysaccharide derived from chitin, which is a linear polymer present in the exoskeletons of crustaceans, insects, fungal cell walls, and plankton^13–15^. The utilization of silk fibroin, gelatin, or calcium phosphate in conjunction with titanium films has been employed to fabricate bioactive coatings^16,17^. It is suggested that the chitosan cationic charge facilitates the attachment of osteoblasts rather than fibroblasts, providing a very effective method for preventing the creation of a fibrous tissue capsule surrounding medical implants.

Pectin, a plant-based polysaccharide, gives strength to the cell walls of higher plants. It is involved in critical cellular activities such as water retention, structural growth, and fruit ripening^18^. Their composition mimics the polysaccharides found in the extracellular matrix of mammals, facilitating particular cellular adhesion^19^. There is a lack of information regarding the impact of pectin-derived polysaccharides on the process of osteoblast development and mineralization^20^. Polyvinyl alcohol (PVA) is a cost-effective artificial polymer that possesses biodegradable properties. Recently, there has been a notable focus on PVA as a polymer that is non-toxic, biocompatible, and biodegradable. This has been particularly relevant in the field of biomaterial and biomedical applications^21^.

Ideal implants possess excellent mechanical and corrosion resistance properties. To achieve superior surface characteristics, osteoconductive, biocompatible, and polymer-based coatings with antimicrobial properties extend the implant's lifespan. Surface modification of materials is essential for enhancing the performance of bulk materials like metals, metal alloys, polymers, and ceramics. Various coating techniques include physical vapor deposition, chemical vapor deposition, electrohydrodynamic methods, electrochemical deposition, spin coating, dip coating, sol–gel coating, electrodeposition, and electroless deposition^22^.

In implant dentistry, nanocoating composites are advanced materials engineered to enhance the surface properties of dental implants. These coatings incorporate nanoparticles and other nanostructures to improve the biological and mechanical performance of the implants. The primary objectives of using nanocoating composites include enhancing osseointegration, providing antimicrobial properties, and increasing corrosion resistance^23^. Nanotechnology exhibits considerable promise in enhancing the quality of implants via innovative interface manipulation and design^24^. It includes surface etching and patterning, layer-by-layer assembly and surface functionalization through doping elements, electrochemical deposition, plasma spraying deposition, pulsed laser deposition, physical vapor deposition, reverse polarization anodization, and surface coating techniques, including electrohydrodynamic techniques and ion-sputtering for deposition at the micro and nanoscale^25,26^. Each technique possesses distinct advantages for achieving the fabrication of a composite layer with a nanotextured surface^27^.

The present study is designed to evaluate the electrospinning/spraying coating on CpTi substrates. The coating solution was prepared from a mixture of chitosan, pectin, and synthetic polymer PVA. To the author's knowledge, this study might be the first to explore the combination of these three mixed natural and synthetic polymers as a nanocoating for dental implants. This polyelectrolyte composite coating was applied to improve the surface of titanium implants, enhancing their mechanical and topographic characteristics as well as their biocompatibility properties. Additionally, the coating aimed to enhance the antibacterial performance, leading to improved bone healing and integration.

## Material and methods

### Materials and sample preparation

**Figure 1:**
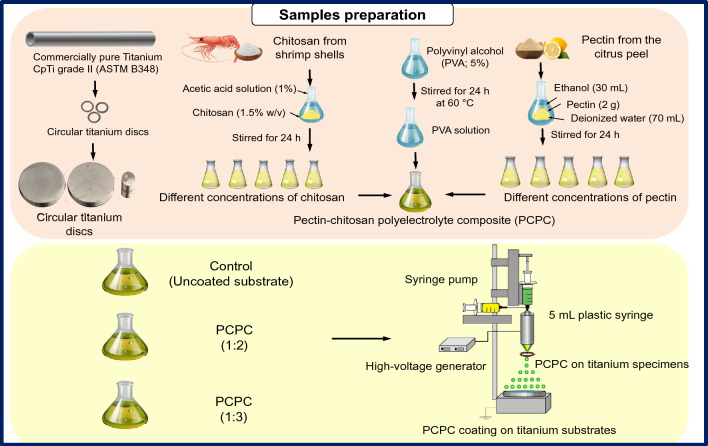
Experimental setup of the study.

Pectin, chitosan, and PVA were purchased and prepared as per our previous study^27^. The experimental design utilized in the fabrication of pectin/chitosan/PVA polyelectrolyte composite (PCPC) solutions is illustrated in Fig. 1. All methods were performed in accordance with the relevant guidelines and regulations. The solution of the coating was prepared from a mixture of chitosan (extracted from shrimp shells ≥ 75% deacetylated, medium M_w_ 190–310 kDa), pectin (extracted from citrus peel-galacturonic acid ≥ 74.0% dried basis, average M_w_ 485 kDa), and synthetic polymer PVA (98% hydrolyzed, average M_w_ 13–23 kDa). As depicted in Table 1, two ratios (PCPC, 1:2 and PCPC, 1:3) were used for this investigation. These optimal ratios were following the results obtained previously^28^. The CpTi grade II discs (18, 10, and 6 mm in diameter and 2 mm in thickness) were selected as the substrate for the coating procedure for each intended investigational test used. The titanium rod was sectioned into discs with the aid of a wire-cutting machine (model DK 7735 from China), following ASM standard^29^. The discs were then smoothed using silicon carbide (SiC) abrasive paper with a 500# grit. Subsequently, the discs were thoroughly cleaned with 96% ethanol (Scharlau, Spain) in an ultrasonic cleaner for 20 min, repeated twice, before being set aside to air dry at room temperature. The different discs’ diameters were selected based on the specific requirements for each conducted experimental test.

**Table 1:** Preparation and combination of the three solutions.

Groups	Pectin (mL)	Chitosan (mL)	PVA (mL)	Ratio
PCPC (1:2)	20	40	30	1:2
PCPC (1:3)	15	45	30	1:3

### Electrospinning/spraying of pectin–chitosan polyelectrolyte composite coating

The system for electrospinning/spraying includes a high-voltage generator (Ya-Feng Technology Co., Taiwan), which was adjusted to 20 kV. Solutions for the process were stored in a 5 mL plastic syringe, which was connected to a 25-gauge stainless steel needle. This syringe, loaded with polymer solution, was then mounted on a syringe pump. The needle was positioned 10 cm away from the collector, and the solution’s flow rate was regulated at 1 mL/h. The procedure was performed at room temperature in a transparent sealed container with a relative humidity range of 40–60%. The produced PCPC coating on titanium samples was left to stand for 24 h under ambient conditions to allow for the evaporation of any residual acid and water before further testing.

### Cytotoxicity test

The MTT assay which measures cell proliferation and cytotoxic effects, is utilized to assess cell metabolic activity^30^. The formazan is dissolved by solubilization agents that are subsequently added, enabling the measurement of the resultant-colored solution via spectrophotometry^31^. There is a direct correlation between the quantity of alive cells present and the intensity of the color^32^. The cytotoxic effect of coated substrates with PCPC (1:2) and PCPC (1:3) in comparison to the uncoated CpTi substrates (control group) was assessed indirectly using the MTT assay. The evaluation focused on isolated fibroblast-like cells and adhered to the standard protocols (ISO 10,993-5:2009) for biological evaluation of medical devices^33^.

A standard MTT solution was stored at − 20 °C away from light for future use, or at 4 °C if it was to be used immediately^34^. The test was conducted using degradation extracts from coated disc samples with PCPC (1:2) and (1:3) and uncoated CpTi disc samples. Seventy-two discs (each 6 mm in diameter and 2 mm in thickness) were further subdivided into three subcategories based on their immersion duration in Dulbecco’s Modified Eagle Medium (DMEM)^35^. Each subgroup was immersed in 5 mL of DMEM to collect the extracts released from the 6 mm disc surface into the culture media. The extracts containing the degradation by-products were collected within three days^36^. Fibroblast-like cells were cultured in a DMEM medium. When the cells reached confluence, they were detached using 0.25% trypsin, and the obtained cells were further sub-cultured^37^.

### Antibacterial susceptibility test

The inhibition zone diameter (IZD) was utilized to determine the antibacterial activity of uncoated CpTi substrates and substrates coated with PCPC (1:2) and (1:3), in accordance with the Kirby–Bauer disk diffusion method^38^. In this experiment, total anaerobic bacteria extracted from the deepest pockets of peri-implantitis served as the model microorganisms. The subjects of this study were recruited from the patient population. The Research Ethics Committee of the College of Dentistry, University of Baghdad granted approval for the current investigation with the approval reference number of 790-2023. All methods were performed in accordance with the relevant guidelines and regulations. All participants were provided with thorough information pertaining to the objectives of the study, and their informed consent was obtained prior to the initiation of sample collection. Subgingival samples were collected from ten systemically healthy individuals, comprising both males and females, aged between 40 and 60 years. These subjects showed symptoms of chronic peri-implantitis, including a probing pocket depth of ≥ 10 mm and a clinical attachment loss ranging from 1–2 mm. Specimens were meticulously obtained from the most profound region of the peri-implant pocket and were subsequently immersed in PBS. The collected samples were immediately transferred to the laboratory for bacterial cultivation. Prior to being inoculated onto a blood agar medium, these specimens were vortexed with a saline solution^39^. The specimens were then subjected to anaerobic incubation for a duration of 48 h. Muller Hinton agar plates were inoculated with a thin film of bacteria using 10 μL of bacterial suspension containing around 1.5 × 10^8^ CFU/mL. Substrates of 6 mm diameter, both coated and uncoated CpTi, were carefully placed at the center of the inoculated plates and incubated anaerobically at 37 °C for up to 72 h before measuring IZD.

### Analytical methods

In order to assess the adhesive strength of coated substrates, the adhesive epoxy tester (Posi Test AT-M model, USA) was employed to conduct the pull-off adhesion test^40^. The test was performed on ten specimens in each coated disc, with PCPC (1:2) and PCPC (1:3). The test standard was accommodated by each disc, which had a diameter of 100 mm and a thickness of 30 mm (Shaanxi Yunzhong Metal Technology Co., Ltd., China). The coated discs were subjected to testing in accordance with the ASTM D4541 standard^41^.

Atomic force microscopy (AFM) was utilized to assess surface characteristics, roughness, topography, and the distribution of particle size. The average surface roughness (*S*_*a*_) and the developed interfacial area ratio (*S*_*dr*_) of each specimen were measured by the AFM with a scan area of 10 µm × 10 µm (NaioAFM 2022 model, Nanosurf AG, Switzerland) for 10 specimens in each group. The AFM probe used for measurements in tapping mode had a gold reflective coating on the tip side of the cantilever (Tap190GD-G), and the detector side was coated with 70 nm of gold.

Field-emission scanning electron microscopy (FESEM; InspectTM F50, FEI USA) was employed to evaluate the coating thickness across the coated groups, ascertain the phases and nano-fibers/particles diameter, and analyse the surface topography and morphology of both uncoated and coated samples. Before examining the morphologies of each sample at varying magnifications, the samples were coated with gold using a compact evaporation vacuum equipped with a gold sputter machine.

Energy dispersive X-ray spectroscopy (EDS) is employed to ascertain the elemental composition of materials. When the electron beam from the FESEM interacts with the sample, it emits characteristic X-rays which are then detected and analyzed by the EDS system. In order to determine the material's elemental composition, quantify the weight percentages of the elements, and map these elements, EDS analyses were implemented^42^.

Fourier transform infrared (FTIR) spectroscopy was utilized to investigate the surface chemical composition of the main materials involved in the study, which were pectin, chitosan, and PVA, as well as the PCPC coating of groups (1:2) and (1:3) specimens. The determination of whether the coating underwent chemical bonding or merely physical blending was ascertained using the FTIR spectrometer (Compact FTRI Spectrometer, Alpha II Bruker, Germany). Measurements were taken within the mid-infrared spectrum range (400–4000 cm^−1^) with a resolution of 4 cm ^−1^. Spectral analysis was performed with OMNIC software, with peak absorption related to particular chemical groups by integrating scholarly references and the software’s library search feature. The spectral information from the FTIR analysis offered a distinctive molecular profile of the sample, which allowed for the accurate determination of the various functional groups within the composite surface coating^43^.

### Statistical analysis

The statistical analysis was performed using Statistical Package for Social Science (SPSS version 27, Chicago in press, Illinois, USA). The results were reported as means and standard deviations. For the bar charts graphical illustration, GraphPad Prism software (GraphPad Software, version 8.0.2, Inc., USA) was used.

## Results

### Cytotoxicity analysis

**Figure 2:**
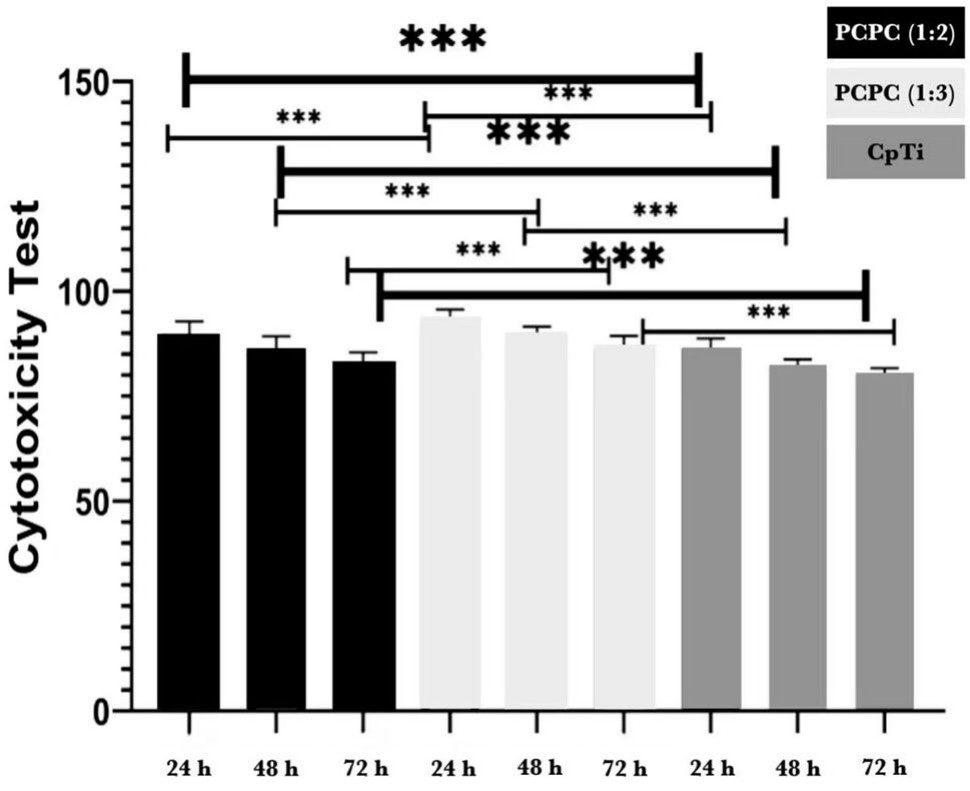
Cytotoxicity test results of the experimental groups after 24, 48, and 72 h.

**Figure 3:**
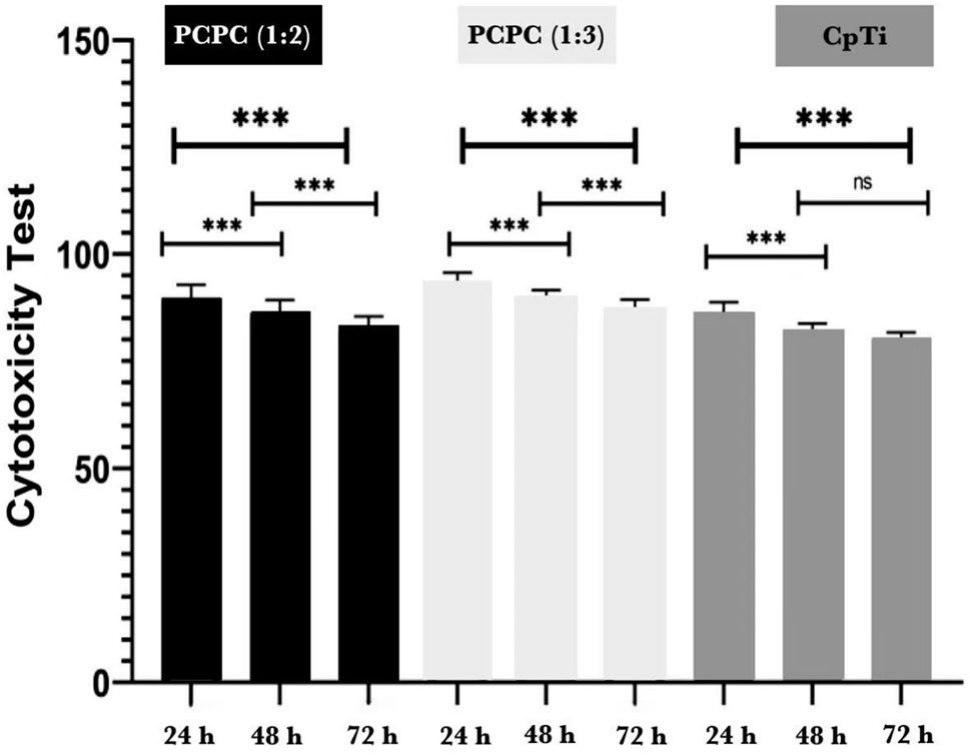
Cytotoxicity test results of the experimental groups after 24, 48, and 72 h.

As depicted in Fig. 2, the cytotoxicity test was performed on human fibroblast-like cells cultivated in DMEM media after 24, 48, and 72 h of exposure on three distinct groups of uncoated commercially pure titanium discs and coated substrates with PCPC (1:2) and PCPC (1:3). The CpTi group had a significantly greater impact on cell viability than all three groups at all time points (*P* < 0.001). At each time point, the mean cell viability of group PCPC (1:3) was the highest, followed by group PCPC (1:2) and CpTi, indicating a dose-dependent cytotoxicity pattern. The results were highly reproducible, as evidenced by the relatively low standard deviation values of all groups. The result of the multiple pairwise comparisons of mean cell viability between groups over time using Tukey HSD, as illustrated in Fig. 3. The results showed that the mean cell viability of each group significantly differed from 24 to 48 h and 72 h, as evidenced by the *P*-values. Nevertheless, the mean cell viability of the CpTi group did not exhibit a significant difference between 48 and 72 h. The mean cell viability of PCPC (1:2) and PCPC (1:3) differed significantly between 48 and 72 h. These results indicate that cell viability normally decreased over time for all groups, suggesting that the materials were biocompatible and did not cause cytotoxicity. The CpTi group had the lowest mean cell viability at all time points, indicating that it was the least biocompatible among the three groups. PCPC (1:2) and (1:3) had similar mean cell viability at 24 h and 48 h, however, PCPC (1:3) had a higher mean cell viability at 72 h, indicating that it was the most biocompatible among the three groups.

### Antibacterial susceptibility

**Figure 4:**
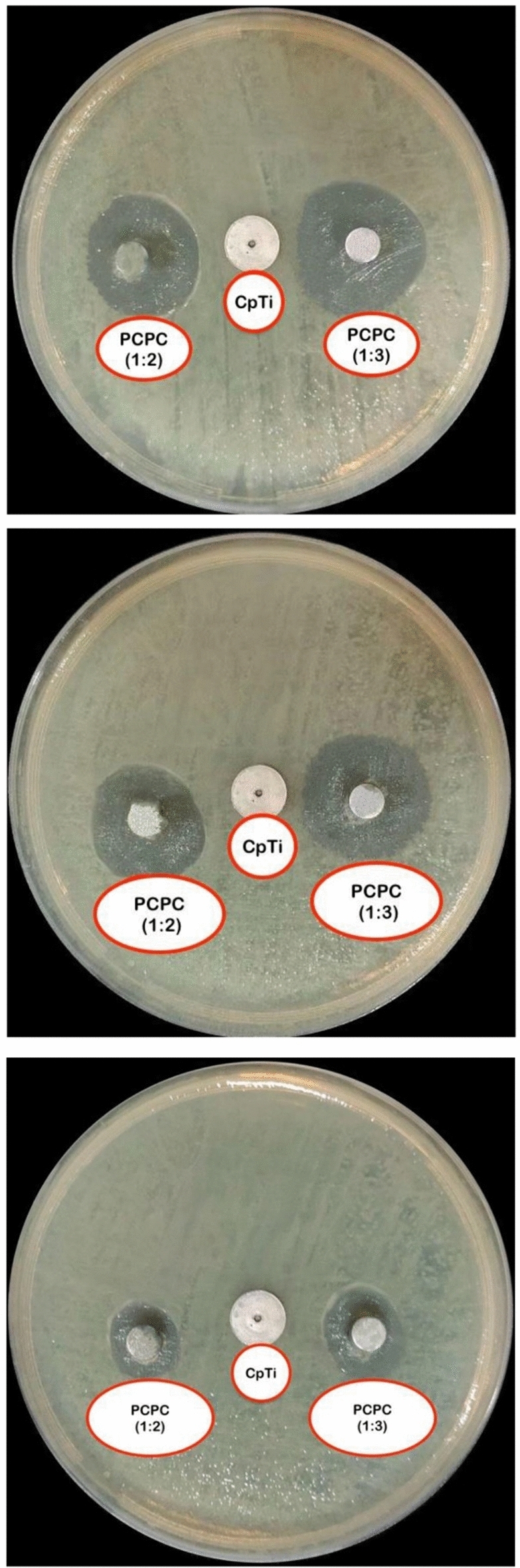
Illustration of antibacterial test of the investigated groups over 24, 48, and 72 h.

**Figure 5:**
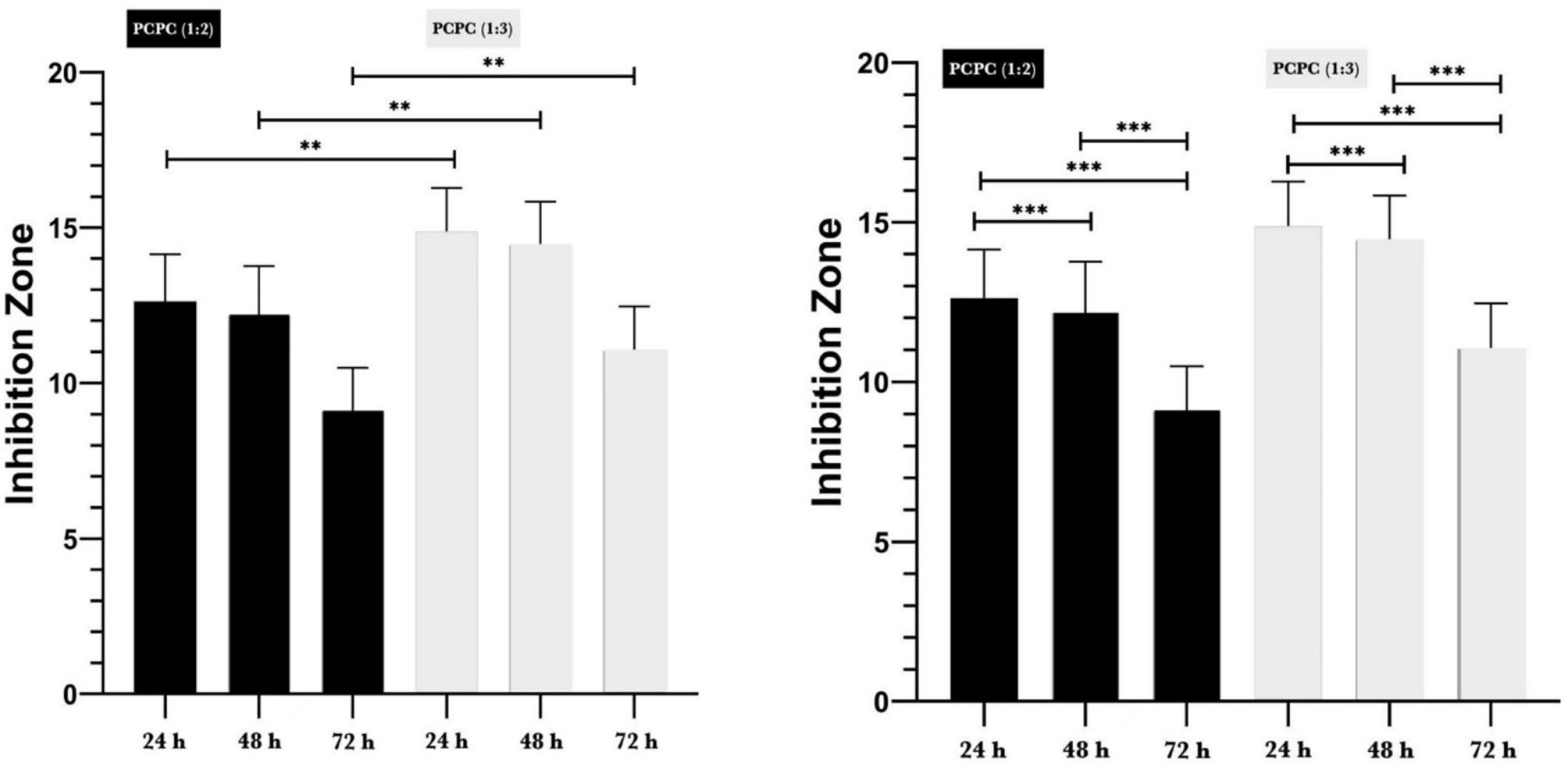
Results of antibacterial test of the coated PCPC (1:2, and 1:3) groups after 24, 48, and 72 h.

The results of the antibacterial susceptibility analysis for the two groups are depicted in Fig. 4. The average IZD was determined at 24, 48, and 72 h following the incubation period. The IZD of PCPC (1:3) was significantly higher than that of PCPC (1:2) at all time points (*P* < 0.01), indicating that PCPC (1:3) exhibited superior antibacterial activity against anaerobic bacteria. The IZD of both groups decreased over time, suggesting a decreasing antibacterial effect. The results of the multiple pairwise comparisons of IZD among time by groups using Tukey HSD are depicted in Fig. 5. The findings indicated that the mean IZD for both groups decreased significantly in the timeframes (*P* < 0.001). These findings suggest that the antibacterial efficacy of both groups decreased over time, as evidenced by the diminished IZD at 72 h.

### Pull-off adhesion test

**Figure 6:**
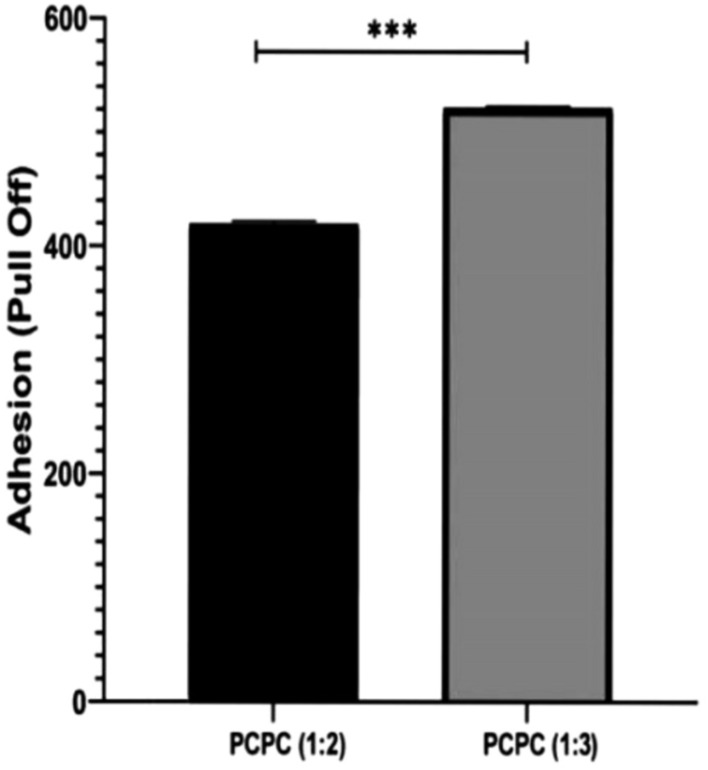
Pull-off adhesion test of the coated PCPC (1:2, and 1:3) groups.

The results of the pull-off adhesion test, which was conducted on the two experimental groups to evaluate the quality of coating adhesion, are illustrated in Fig. 6. The pull-off adhesion of the two compared groups exhibited a statistically significant difference. The experimental coating employed in this group may have enhanced bonding strength in comparison to PCPC (1:2), as evidenced by the substantially higher pull-off adhesion observed in PCPC (1:3).

### AFM analysis

**Figure 7:**
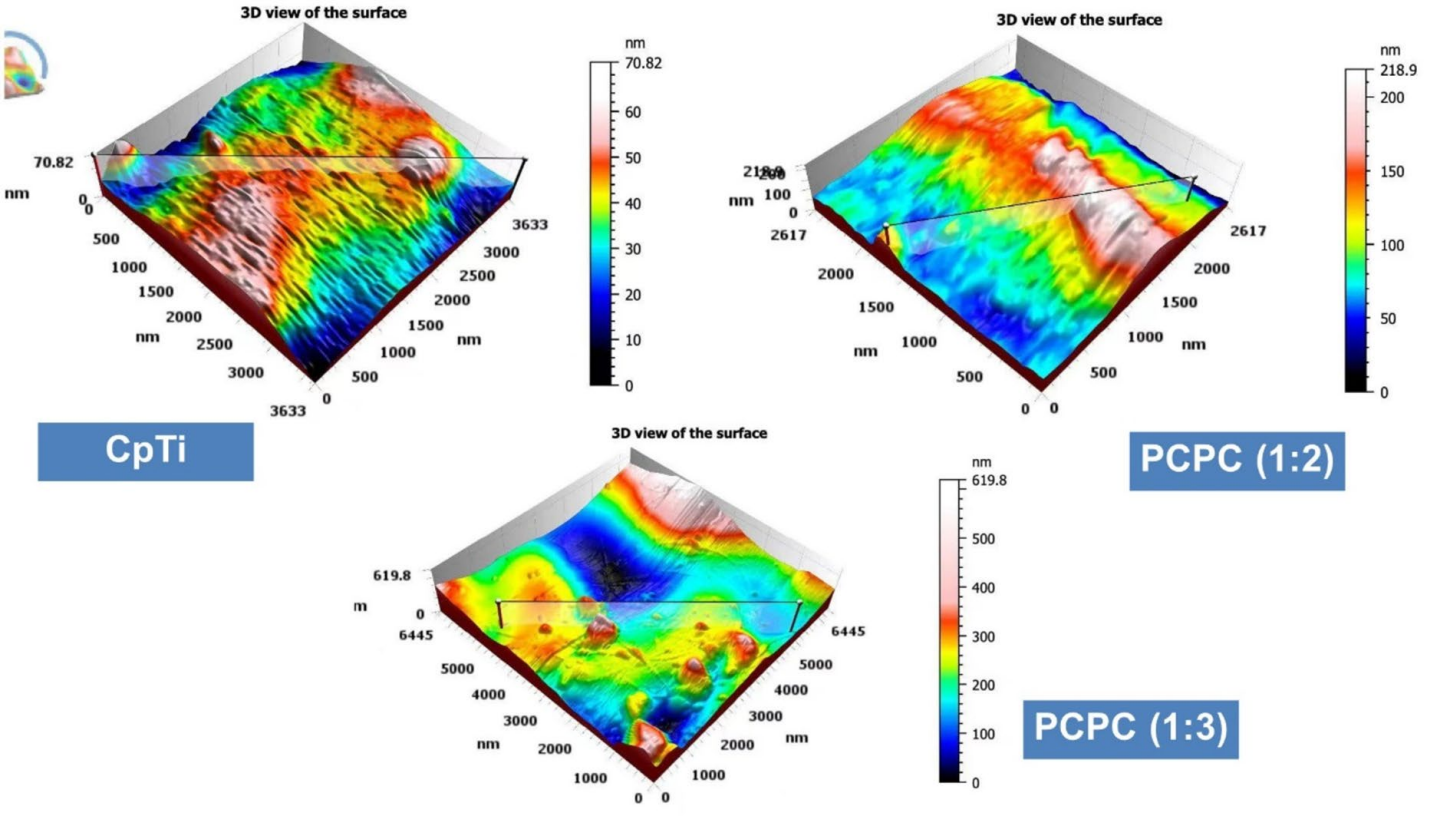
Topographic features illustration of the three experimental groups.

**Figure 8:**
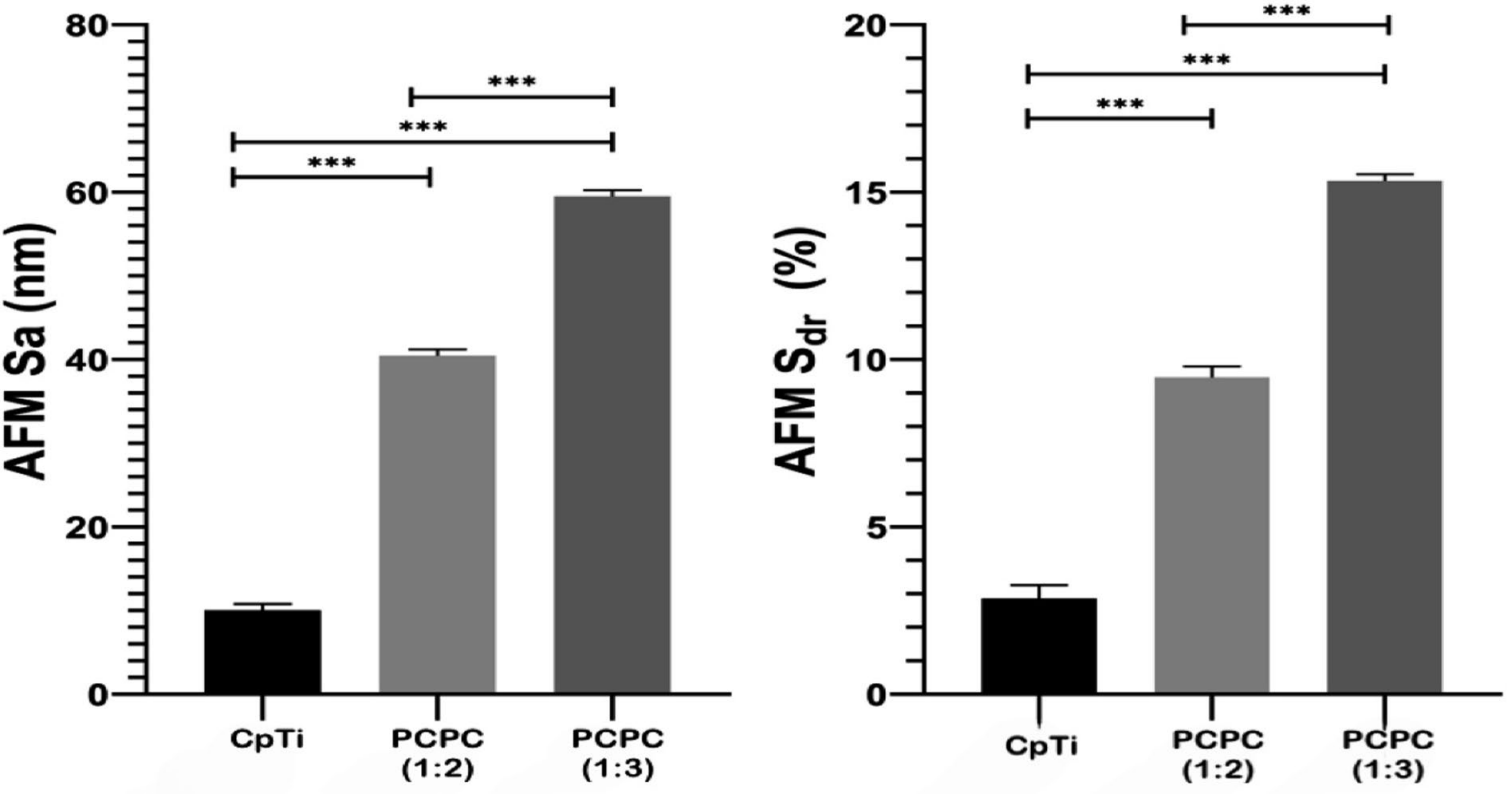
Illustration of the roughness parameters (*S*_*a*_ and *S*_*dr*_) of the experimental groups.

The surface roughness parameters of the samples were measured by AFM and the results are shown in Fig. 7. The mean values of AFM-*S*_*a*_ (arithmetic mean height of the surface) and AFM-*S*_*dr*_ (developed interfacial area ratio) were calculated for each group and compared by one-way ANOVA. The results revealed that there were significant differences among the groups for both parameters (*P* < 0.001). The CpTi group had the lowest values of AFM-*S*_*a*_ and AFM-*S*_*dr*_, indicating the smoothest surface. PCPC (1:3) had the highest values of AFM-*S*_*a*_ and AFM-*S*_*dr*_, indicating the roughest surface. PCPC (1:2) had intermediate values of AFM-*S*_*a*_ and AFM-*S*_*dr*_, indicating a moderate surface roughness. The ANOVA results showed that there were significant differences among the three groups for both the AFM-*S*_*a*_ and AFM-*S*_*dr*_ parameters (*P* < 0.05). The Tukey HSD test revealed that coated groups had significantly higher AFM-S_a_ and AFM-*S*_*dr*_ values than CpTi, indicating that the coated groups had more rough and textured surfaces (Fig. 8). PCPC (1:2) also had significantly lower AFM-*S*_*a*_ and AFM-*S*_*dr*_ values than PCPC (1:3), indicating that PCPC (1:3) revealed better nano-roughness parameters.

### FESEM and EDS analysis

**Figure 9:**
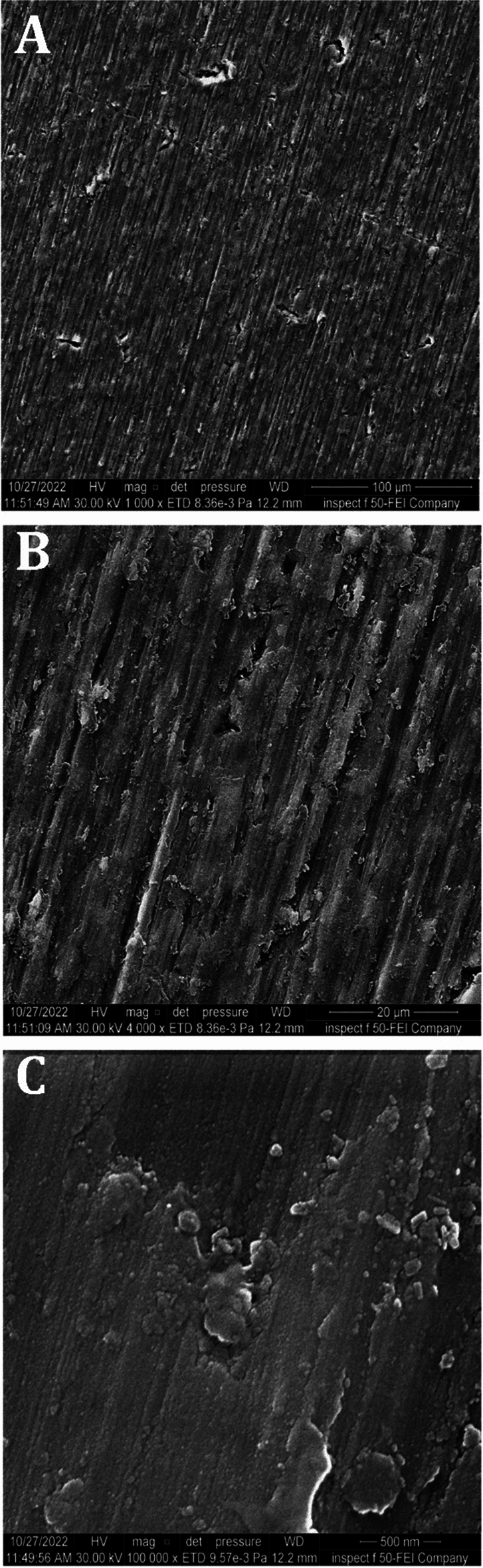
FESEM images analysis of CpTi substrate, (**A**): at 1000×, (**B**): at 4000×, (**C**): at 100,000×.

Figure 9 illustrates the morphological FESEM surface analysis images of a CpTi disc. The images demonstrated that the CpTi substrate exhibits a uniform surface with a pattern of multiple peaks and valleys, and no defects or fractures were visible. The SiC grinding process is the direct cause of the indentation surface characteristics.

**Figure 10:**
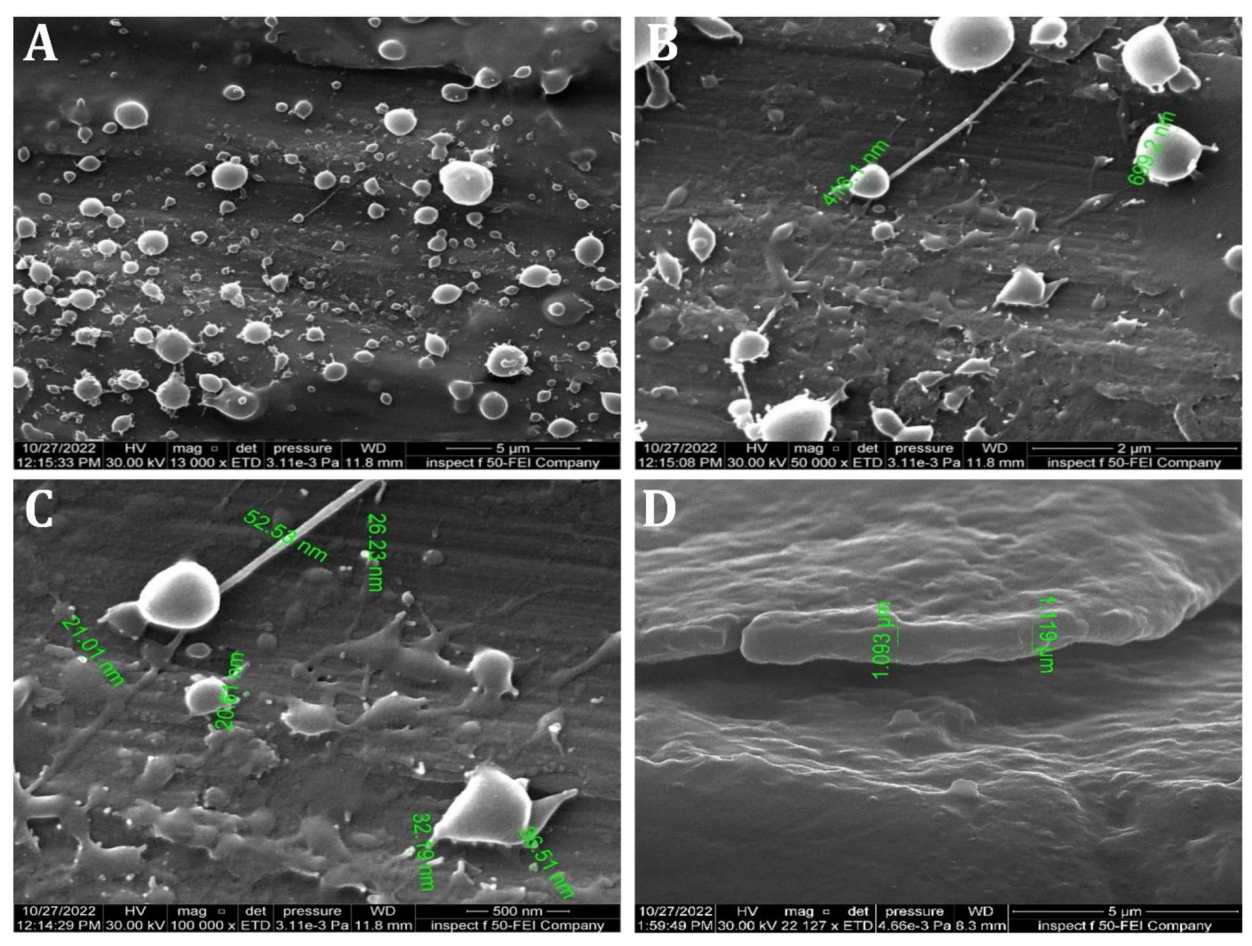
FESEM images analysis of PCPC (1:2) coated CpTi substrate, (**A**): At 13,000×, (**B**): At 50,000×, (**C**): At 100,000×, (**D**): Cross-sectional image at 22,127×.

The morphological FESEM images of coated CpTi substrates with PCPC (1:2) are depicted in Fig. 10. The coat is uniformly deposited on the CpTi substrate surface. Nano-sized minuscule structures are interspersed with both small and large spherical particles, and the coating layer is free of fractures. The diameters of these spherical particles range from 21 to 699 nm (Fig. 10A–C). The substrate surface contains nano-sized particles that are dispersed. Figure 10D illustrates that the coating layer is adhered to the titanium surface with a cross-sectional thickness of approximately 1 µm.

**Figure 11:**
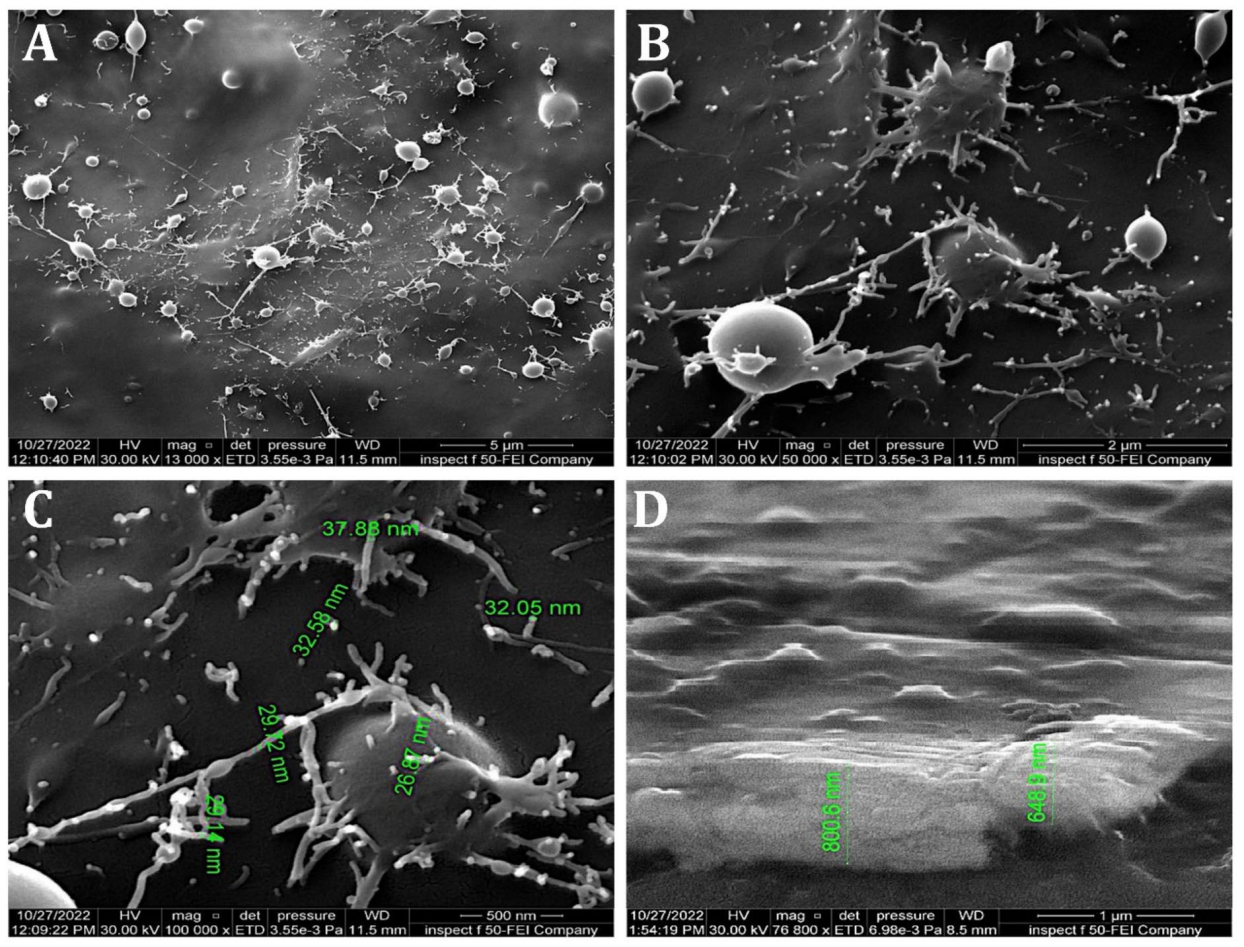
FESEM images analysis of PCPC (1:3) coated CpTi substrate, (**A**): At 13,000×, (**B**): At 50,000×, (**C**): At 100,000×, (**D**): Cross-sectional image at 76,800×.

The morphological FESEM images of coated CpTi substrates with PCPC (1:3) are illustrated in Fig. 11. The images depict a coating that is consistently applied to the CpTi substrate surface, resulting in a closely interwoven network pattern. The coating is composed of nano-sized fibers with minuscule spherical particles and a few large nano-particles that are highly adhered together and free of any cracks. These nanostructures have a diameter that varies from 26 to 37 nm (Fig. 11A–C). The fibers and particles’ aggregation and clustering are more uniform than in PCPC (1:2), suggesting a more cohesive structure. The coating layer is uniformly deposited on the substrate, with a cross-sectional thickness of less than 1 µm (Fig. 11D).

**Figure 12:**
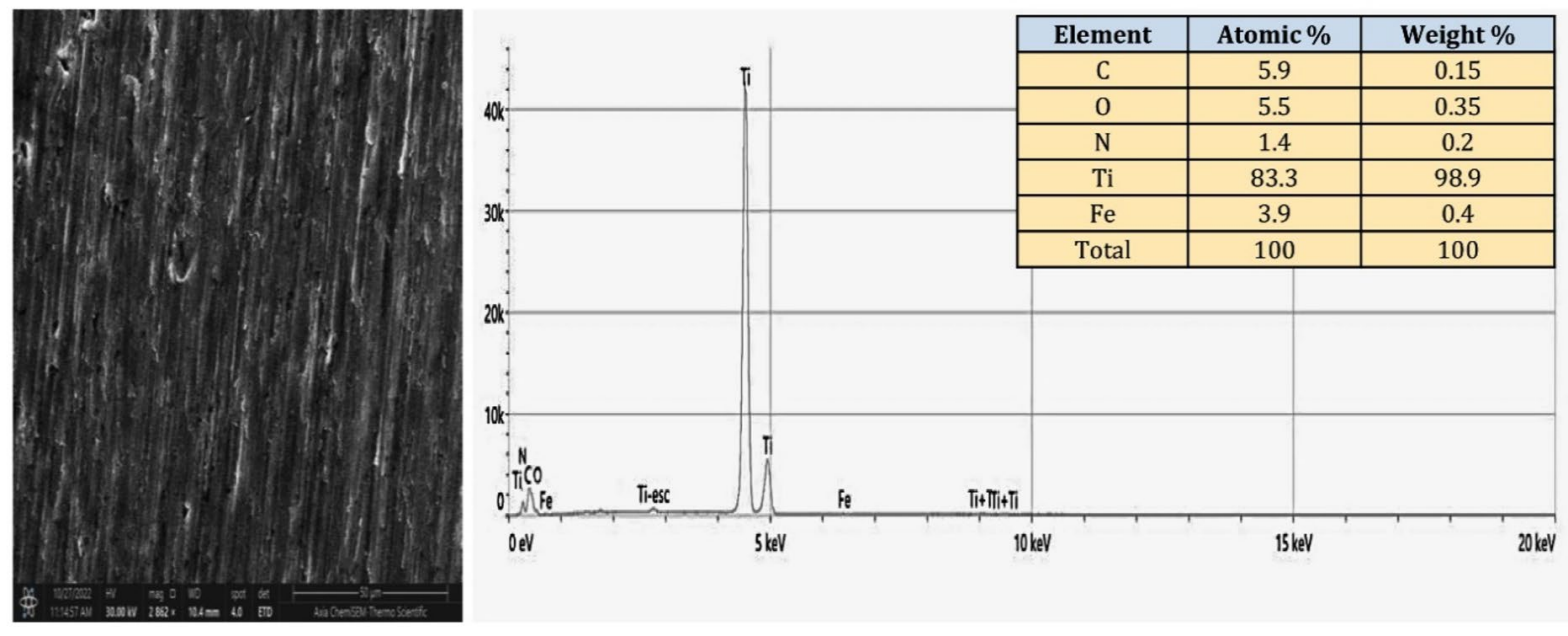
EDS spectrum and elemental analysis of the control (CpTi) group.

**Figure 13:**
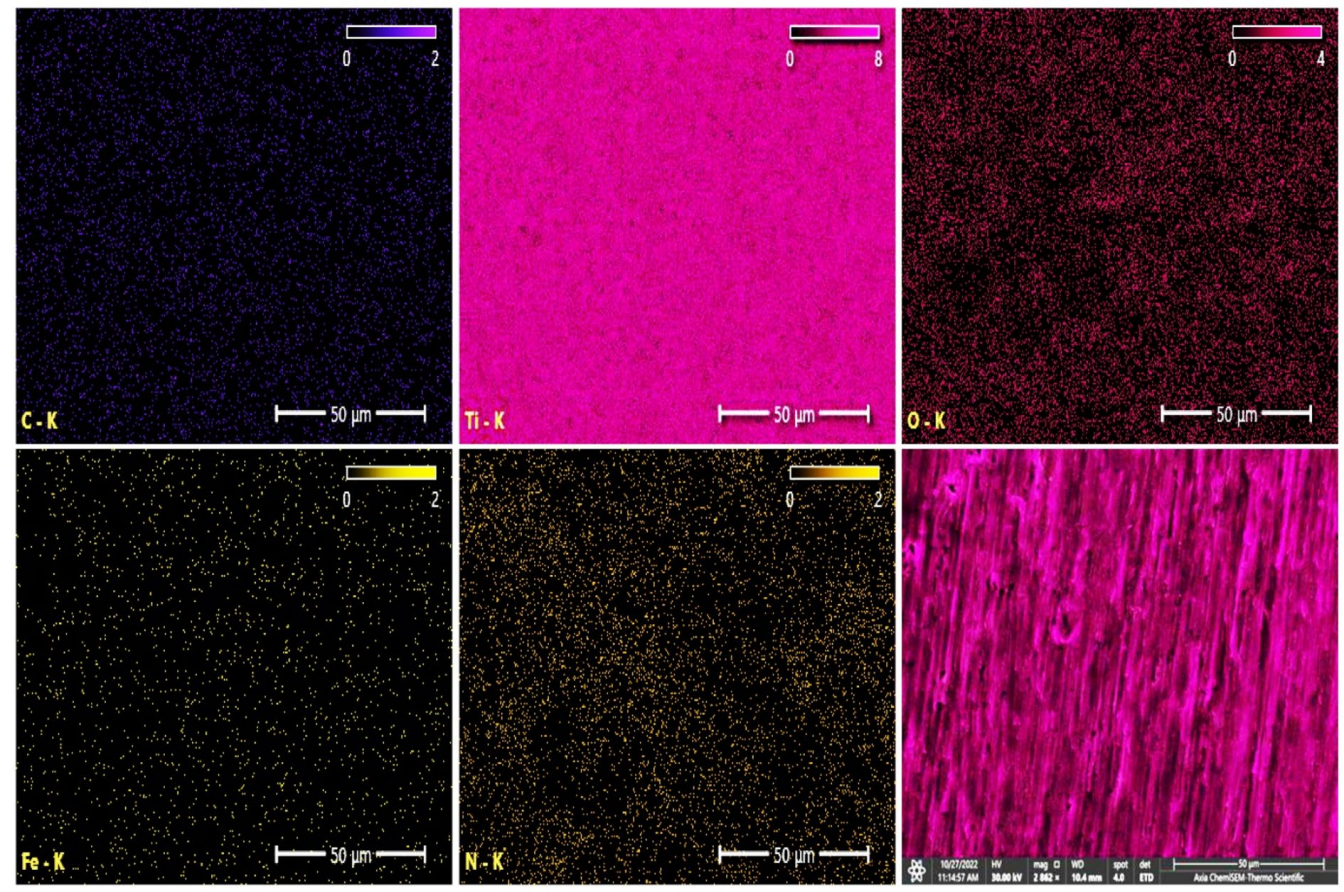
Elemental mapping of the control (CpTi) group.

Figure 12 illustrates the EDS spectrum and elemental analysis of the CpTi substrates, while Fig. 13 illustrates the elemental mapping. The EDS spectrum image suggests the presence of a peak energy at 4.5 keV that is apparent predominant for the titanium element (98.9 wt%). The carbon element (0.15 wt%) peak is observed at 0.25 keV, while the oxygen element (0.35 wt%) peak is observed at 0.5 keV. At 0.75 keV, a low energy level is indicative of a low content of N and Fe elements.

**Figure 14:**
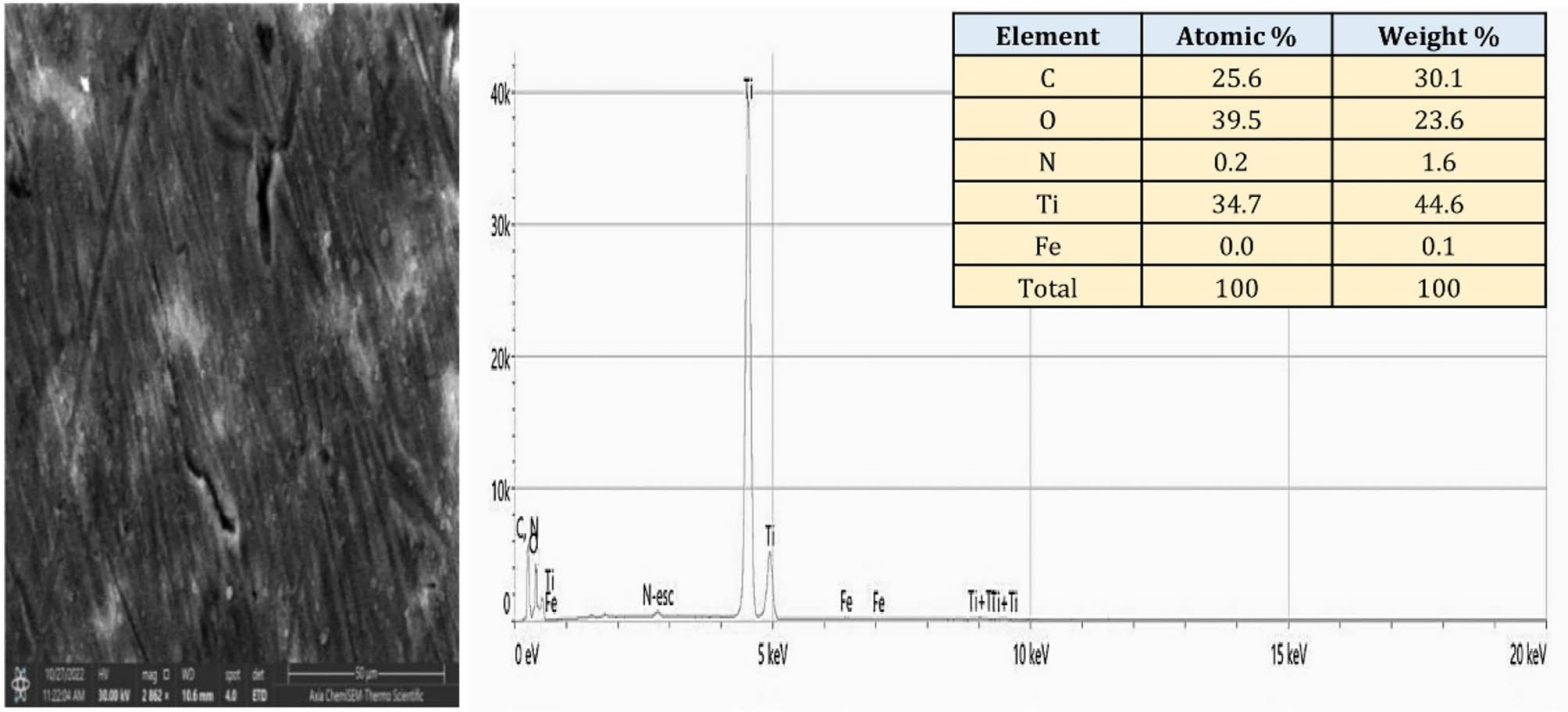
EDS spectrum and elemental analysis of PCPC (1:2) coated substrates.

**Figure 15:**
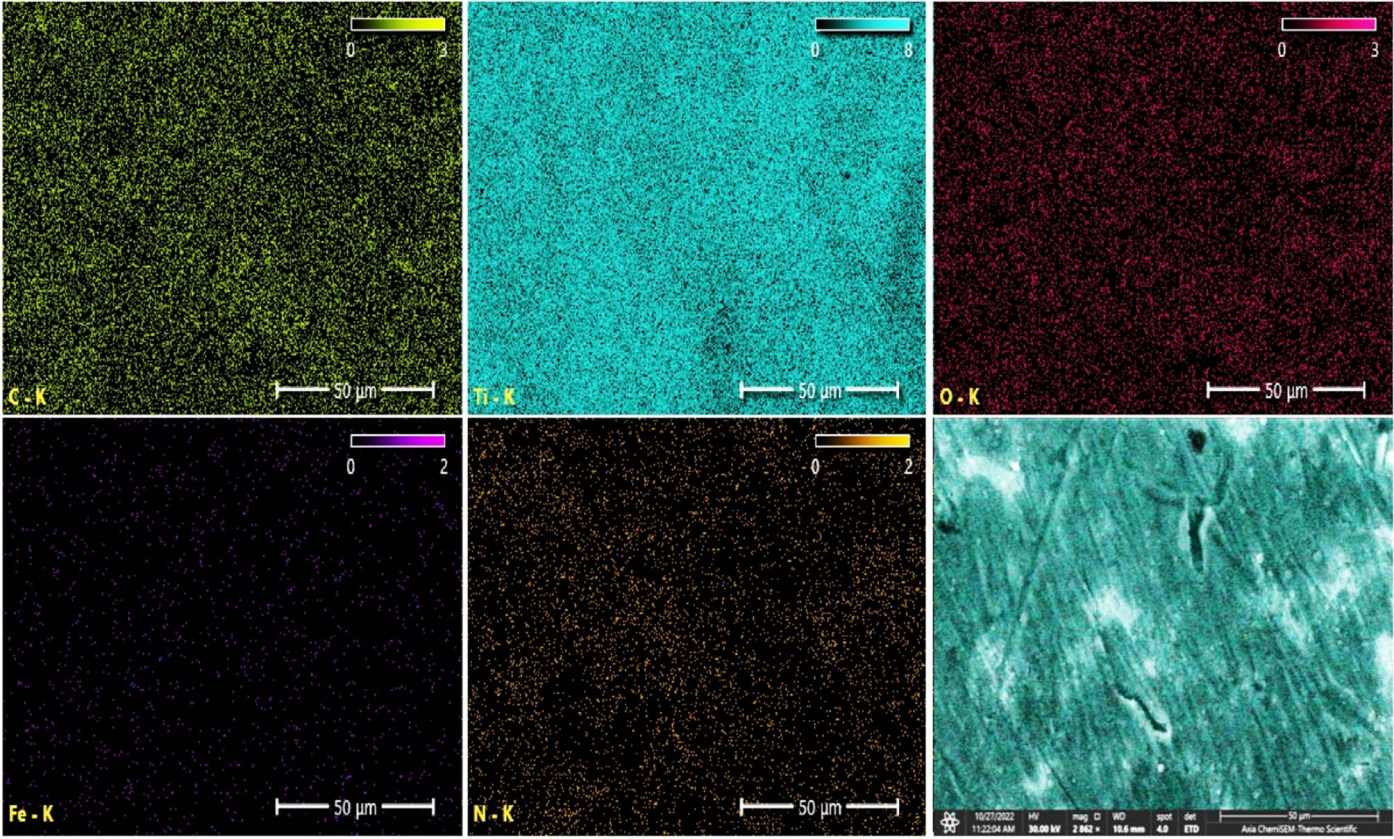
Elemental mapping of the PCPC (1:2) coated substrates.

Figure 14 presents the EDS spectrum and elemental analysis of the PCPC (1:2) coated CpTi substrates, while Fig. 15 displays the elemental mapping. The EDS spectrum image reveals the presence of strong peaks of carbon (30.1 wt%) and oxygen (23.6 wt%) at 0.25 keV and 0.5 keV, respectively, which are both chitosan and pectin. At 0.75 keV, there is a significant presence of nitrogen (1.6 wt%), primarily associated with chitosan. The FESEM/EDS mapping pictures, which show a uniform distribution of these elements, confirm the efficient coating on the substrate, with the titanium peak appearing at 4.5 keV with less content (44.6 wt%) compared to the uncoated CpTi substrate.

**Figure 16:**
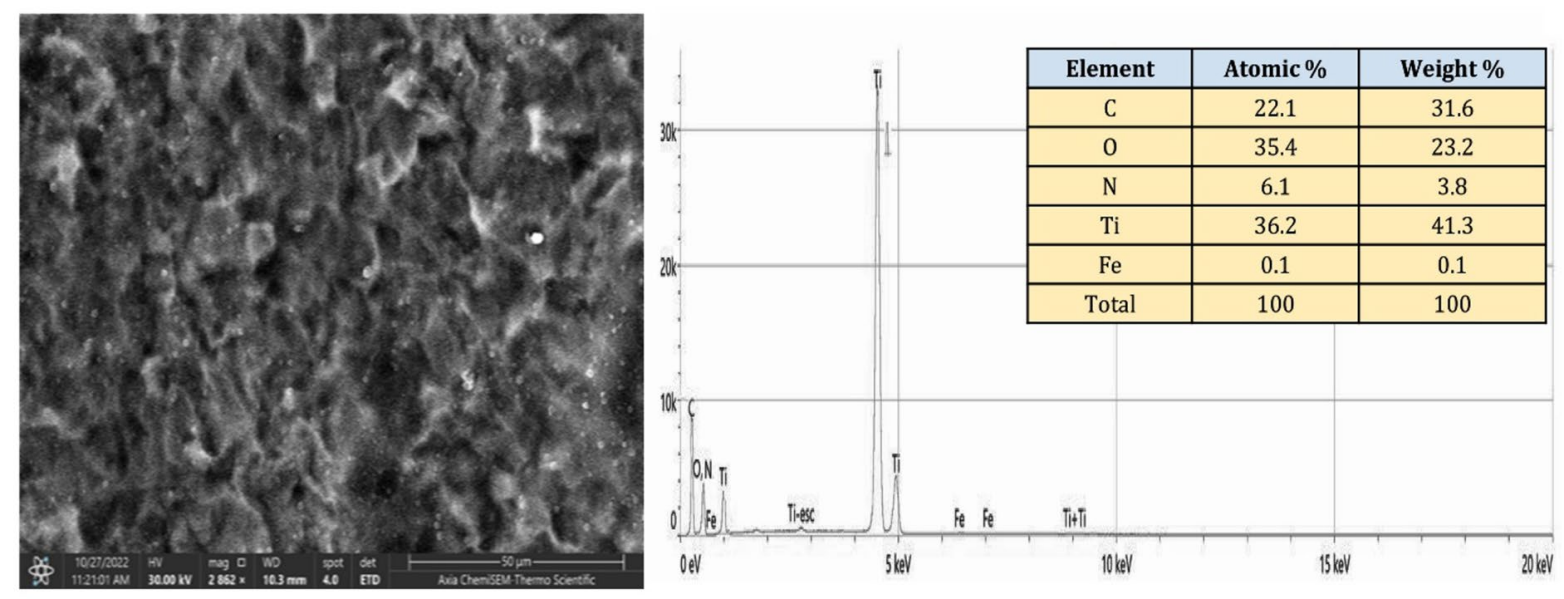
EDS spectrum and elemental analysis of the PCPC (1:3) coated substrates.

**Figure 17:**
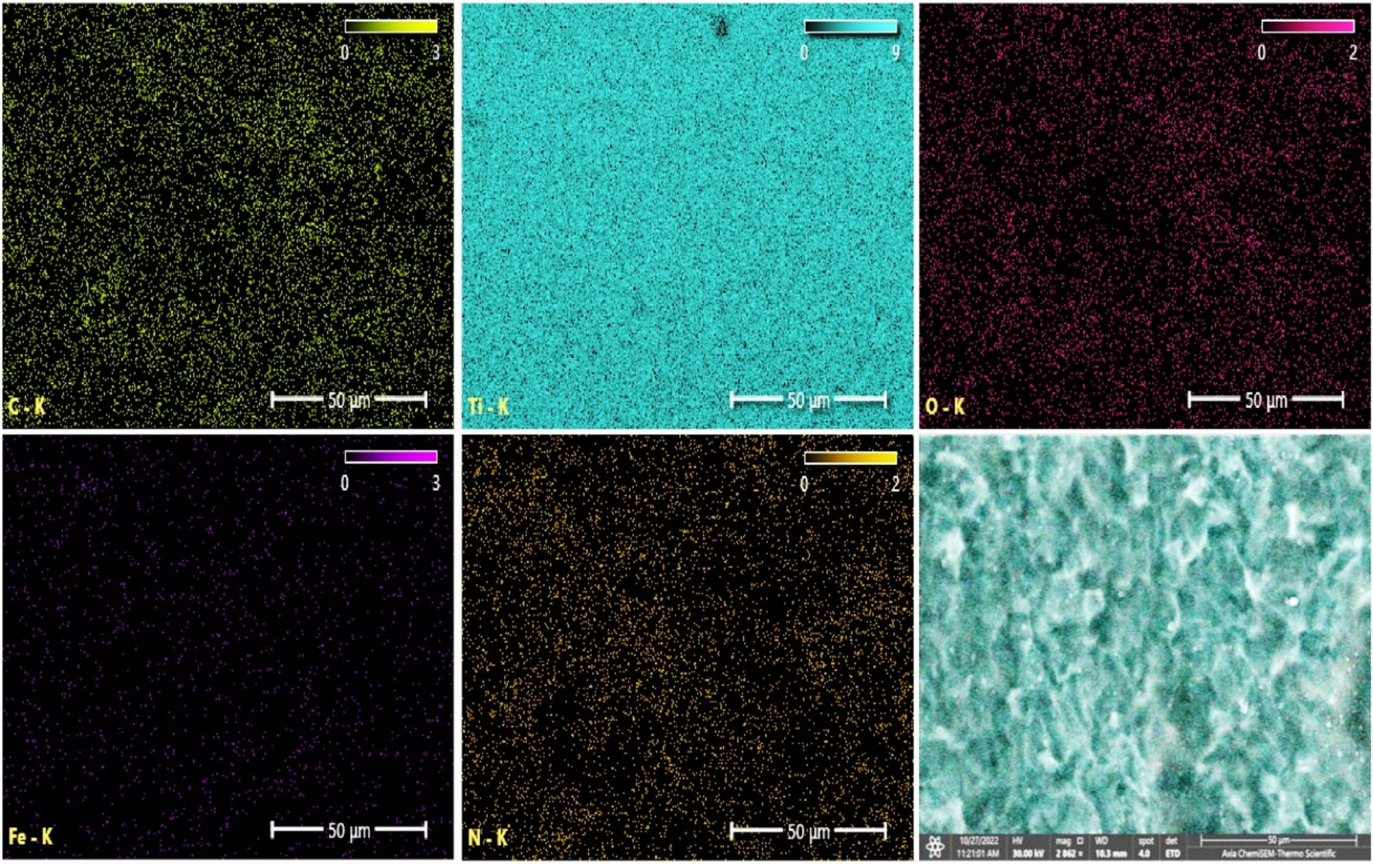
Elemental mapping of the PCPC (1:3) coated substrates.

Figure 16 illustrates the EDS spectrum and elemental analysis of the PCPC (1:3) coated CpTi substrate, while Fig. 17 illustrates the elemental mapping. The EDS spectrum image indicates the presence of robust peaks of carbon (31.6 wt%) and oxygen (23.2 wt%) at 0.25 keV and 0.5 keV, respectively. These peaks are indicative of chitosan and pectin, respectively. At 0.75 keV, a greater quantity of nitrogen (3.8 wt%) is observed, which is primarily due to the increased concentration of chitosan in this group. The FESEM/EDS mapping images demonstrate a uniform distribution of these elements, which confirms the efficient coating on the substrate. The titanium peak appears at 4.5 keV with a lower content (41.3 wt%) compared to the uncoated CpTi substrate.

### FTIR analysis

**Figure 18:**
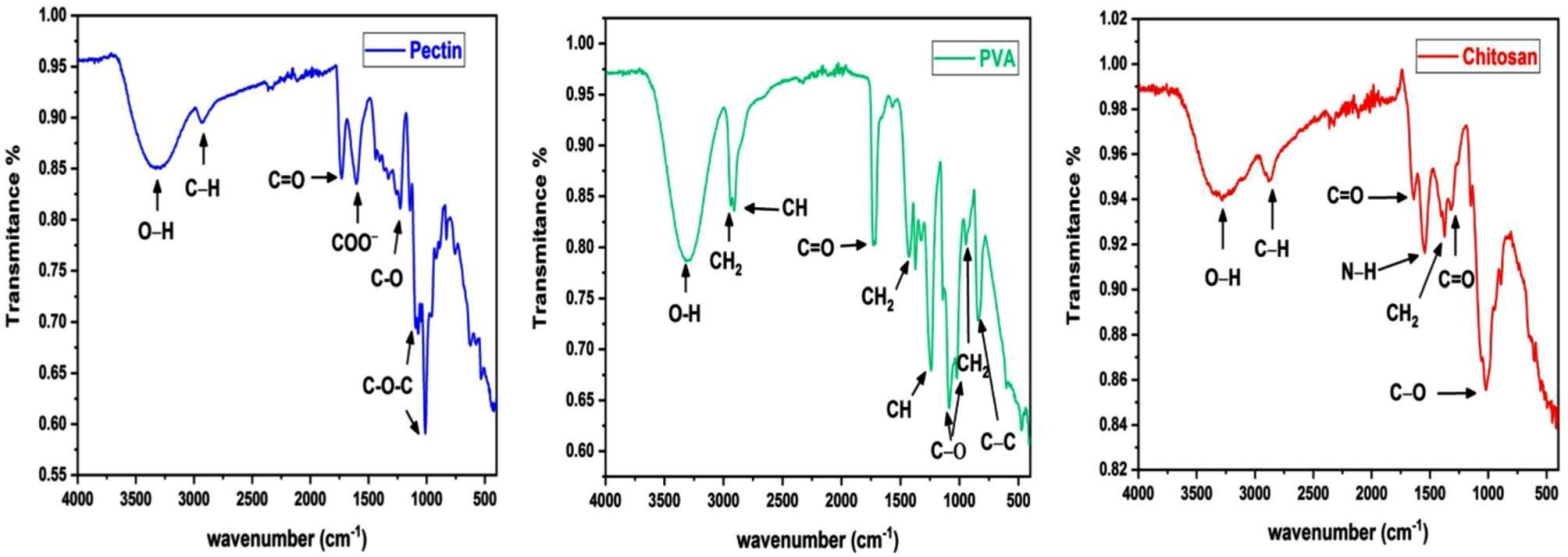
FTIR spectrum of pectin, chitosan, and PVA.

The FTIR spectrum data of the three combined materials is depicted in Fig. 18. The symmetric stretching vibration of the OH- bond is represented by a peak at 3274 cm^−1^ in the FTIR spectrum of chitosan. The C–H stretching bond was represented by the peak at 2874 cm^−1^. The C=O stretching bond was responsible for the peak observed at 1643 cm^−1^. A peak at 1550 cm^−1^ that was attributed to the N–H bond has been determined, whereas the peak at 1366 cm^−1^ was attributed to the C–H bond in CH_2_. The C=O stretching bond was represented by the minuscule peak at 1304 cm^−1^. The O–H bond is the cause of the peak at 3033 cm^−1^ in the FTIR spectrum of PVA. The bands at 2932 and 2901 cm^−1^ are responsible for the asymmetric and symmetric stretching vibrations of C–H in the CH_2_ bond, respectively. The C=O stretching bond is responsible for the observed peak at 1714 cm^−1^. The CH_2_ stretching bond was represented by the peaks observed at 1430, 1240, and 941 cm^−1^. The O–H bond is responsible for the peak at 3308 cm^-1^ in the FTIR spectrum of pectin. The stretching bond of CH_2_ was observed at 2931 cm^−1^. The C=O stretching bond is responsible for the absorption band at approximately 1738 cm^−1^. The asymmetric stretching vibration of the carbonyl group of the carboxylate ion COO- is represented by the peak at 1612 cm^−1^. The C–O stretching bond was observed at 1222 cm^−1^.

**Figure 19:**
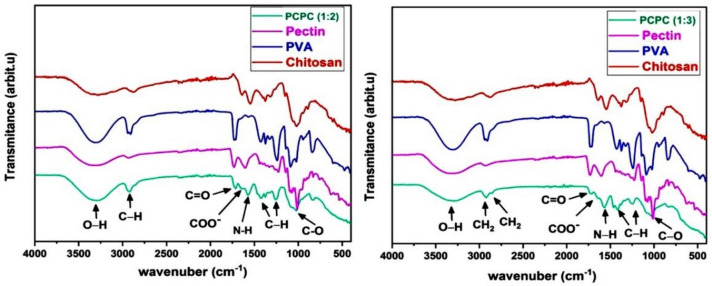
FTIR spectra comparison of the two PCPC groups with pure pectin, chitosan, and PVA.

During the analysis of the FTIR spectrum of PCPC (1:2), which has a pectin-to-chitosan ratio of 1:2, it was compared to the spectra of unadulterated PVA, pectin, and chitosan. The comparison demonstrated that all materials contained existing bonds; however, no new bonds were generated, suggesting that the components were physically combined rather than subjected to a chemical reaction. The presence of the O–H stretching bond at 3299 cm^−1^ is underscored by the FTIR spectral analysis of this group, as illustrated in Fig. 19. Furthermore, stretching bonds of CH_2_ were observed at 2932 cm^−1^. The C=O stretching bond is associated with the absorption band at 1734 cm^−1^. The asymmetric elongation of the carbonyl group in the carboxylate ion COO– is represented by the peak at 1636 cm^−1^. The N–H bond is indicated by a peak at 1565 cm^−1^. Peaks at 1426 and 1246 cm^−1^ are indicative of the elongation of C–H from CH_2_. Additionally, the C–O stretching bond was identified at 1018 cm^−1^.

The FTIR spectrum of PCPC (1:3), which has a pectin-to-chitosan ratio of 1:3, did not reveal any new bonds, indicating that the substances were combined physically without a chemical reaction. The FTIR spectral comparison of group 4 against purified PVA, chitosan, and pectin is illustrated in Fig. 19. At 3315 cm^-1^, the O–H bond is observed. Furthermore, the C–H stretching bond of CH_2_ groups was observed at 2932 and 2852 cm^−1^. The C=O stretching bond is represented by the absorption at 1730 cm^−1^. The symmetric elongation of the carbonyl group in the carboxylate ion COO– is associated with the bond at 1650 cm^−1^. The N–H bond is identified at 1570 cm^−1^, while the C–H stretching bonds are recognized at 1410 and 1250 cm^−1^. Finally, the C–O bond was detected at 1008 cm^−1^.

## Discussion

The objective of this study was to enhance the assessment of the recently developed PCPC for dental implants following electrospraying coating on CpTi. In our earlier study^28^, we assessed several combinations of pectin and chitosan ratios. Through a series of experimental testing, we identified the two most suitable ratios. This study evaluated the compositions of PCPC (1:2 and 1:3). The cytotoxicity test indicated that PCPC (1:3) exhibited a higher mean cell viability after 24, 48, and 72 h of exposure than the other groups. The cellular toxicity response is influenced by a variety of factors, including particle size, form, surface charge, material concentration, and composition^44^. Furthermore, the geometry of nanoparticles influences their interaction with cellular surfaces and their internalization by cells^45^. The clustering, persistence, and interaction with cellular membranes and receptors of nanocoatings can be influenced by their electrical charge on the surface. The ability of the nanocoating to decompose, the compatibility with biological systems, and the potential for the liberation of deleterious ions or compounds are all determined by their chemical composition^46^. This research fabricated a nanocoating using organic natural polysaccharides, specifically pectin, and chitosan. The potential efficacy of these natural polysaccharides in wound dressing applications has been confirmed by their enhanced cell adhesion and survival^47^. Even after 72 h of exposure, the average cell viability of the CpTi group was still above 80%. Clearly, a material be classified as non-cytotoxic if it exhibits a cell viability of over 70% after a 24 h exposure period^48^. Potentially, oxidative stress, inflammation, and cell mortality may result from the leaching of metallic ions into the surrounding biological environment by CpTi^31^. The release of metal ions and the interaction with surrounding cells could be altered by modifying the CpTi surface through the addition of various substances. In order to improve cell survival, it is essential that the coating utilized in PCPC (1:3) either discharge fewer metal ions or exhibit superior biocompatibility properties in comparison to PCPC (1:2). Additionally, the mechanical properties and physical surface of these materials can have a substantial effect on cellular behaviors, including differentiation, growth, and attachment. This study is limited by its failure to quantify the release of metal ions or to consider the antioxidant properties of the materials, as well as its reliance on a specific type of cell and cytotoxicity test^49^. In order to quantify the release of metal ions and ascertain the antioxidant properties of the materials, future research should expand its scope to include cytotoxicity assessments and a variety of cell types. Also, in order to gain a more thorough comprehension of the materials' efficacy, the evaluation should include in-vivo testing with animal models.

In order to ensure the longevity of dental implant materials, it is essential to prevent pathogens from initially adhering to the surface and to promote the proliferation of healthy tissue around the implant. This investigation demonstrated that both experimental groups exhibited exceptional IZD against anaerobic bacteria. On the other hand, PCPC (1:3) demonstrated a substantial disparity from PCPC (1:2) in all three time periods. The results that were observed may be attributed to the increased proportion of chitosan in PCPC (1:3). Coating titanium with organic polysaccharides, such as chitosan, results in antibacterial activity against a wide range of microorganisms, including anaerobic ones^50^. This reinforces the potential of chitosan as a candidate for antimicrobial coatings in a variety of biomedical situations. Chitosan-based coatings have the potential to improve the efficacy of dental and orthopaedic implants^50,51^. Although in-vitro methods offer valuable insights into biological activity, their application in clinical practice is inadequate without an understanding of in vivo biokinetics^52^. Subsequently, animal models continue to be indispensable for future investigations in this domain.

Pull-off adhesion results revealed a significant difference; the mean of PCPC (1:3) coated substrates showed a mean of 519 psi, while PCPC (1:2) coated substrates showed a mean of 419 psi. The significantly higher pull-off adhesion in PCPC (1:3) suggests that the experimental conditions or material used in this group may have provided improved bonding strength compared to the other group. The enhanced adhesion observed in PCPC (1:3) may be attributed to the reduced proportion of pectin present with a higher chitosan ratio. This finding aligns with conclusions drawn from previous research^53^. Additionally, the surface free energy of the substrates is improved by mechanical surface treatment, such as SiC machining, which improves adhesion quality^54^. Surface roughness results in an increased number of contact points at the interface as a result of the broadened interfacial surface area^55^.

The CpTi group had the lowest AFM-*S*_*a*_ and AFM-*S*_*dr*_ values, indicating a smoother surface. PCPC (1:3) had the highest values of AFM-*S*_*a*_ and AFM-*S*_*dr*_, indicating the roughest surface. PCPC (1:2) had intermediate values for AFM-*S*_*a*_ and AFM-*S*_*dr*_, indicating a moderate surface roughness. The surface roughness metrics obtained from AFM provide insights into the samples' topography and texture, which have implications for their biological and mechanical behavior. For instance, a more textured surface may promote better integration between bone and titanium implants, enhancing contact at the interface. Determining the ideal surface texture for titanium implants requires careful consideration of these aspects in conjunction with the specific clinical context. The application of nano-scale topography ranging from 1 to 100 nm is commonly used in implant dentistry^56^. The nanoscale surfaces have complex textures that result in increased surface energy, leading to improved wettability and enhanced cellular adhesion. This can elucidate the reason for the observed elevation in the average cell viability on coated substrates as opposed to uncoated CpTi substrates. Through the improvement of specific protein interactions, nanostructured surfaces have the capacity to facilitate bone cell attachment, proliferation, and differentiation^57^. With AFM measurements, the current study confirmed nano-scale roughness and the developed interfacial area ratio. Compared to other groups, PCPC (1:3) coated substrates showed a remarkably significant increase in both nano-roughness and developed surface area ratio. The more uniform nanofiber/particle topography on the substrates generated with the electrospinning/spraying technique could be the reason for this significant roughness increase^58^. Compared to the control group, the pectin-coated titanium surface increased the surface roughness parameters, including *S*_*a*_ and *S*_*dr*_, which aligns with current findings on how the coated groups enhanced the surface nano-roughness.

The FESEM results of the uncoated CpTi control group exhibit a consistent surface appearance, characterized by a pattern of raised areas and depressions, without any visible imperfections or fractures, which can be attributed to the directional grinding process utilizing SiC abrasive grit. When observed under FESEM, these patterns reveal parallel lines or tracks that align with the direction of the machining process^59^. The uniformity and spacing of these grooves can have an impact on surface mechanical interlocking capability and subsequent coating adhesion quality. The FESEM images are consistent with the AFM analysis findings regarding surface topography and surface roughness. The FESEM pictures of substrates coated with PCPC (1:2) showed that the coating layer, which is mostly made up of small and large spherical nanoparticles, was spread out well. These nanoparticles range in diameter from 21 to 699 nm. The particles disperse and connect in a loose net-like pattern. The coating thickness is approximately 1 µm and adheres to the titanium substrate with no evident crack. The presence of pectin and a lower chitosan ratio within this group may predict enlarged average nanoparticles compared to the other coated group. According to a previous study^60^, adding pectin increases the overall intensity of the polymer solution, which makes the coating spread out over a larger area. The FESEM images of PCPC (1:3) coated substrates showed a coating that is consistently applied to the CpTi substrate surface, forming a closely interwoven network pattern. The coating is free of any cracks and consists of nano-sized fibers with tiny spherical particles and a few larger nano-particles that are highly adhered together. The diameter of nanofibers and particles ranges between 26 and 37 nm. The fibers and particles seem to be clumped together and forming a more even structure than in other coated groups. This suggests a more complex network structure that is important for cell attachment, nutrient transport, and mechanical integrity^61^. The coating layer is well deposited and adhered to the titanium substrate with a cross-sectional measurement under 1 µm in thickness. This finding confirms that this group has the highest adhesion strength, as the coating layer adhered well with no gap, crack, or delamination present that could compromise the coating’s integrity. PVA has been shown to work well with electrospun chitosan polymer, making it easier to spin and forming smooth fibers^62^. The FTIR spectrum showed bands that were made up of functional groups like O–H, CH_2_ stretching, C=O stretching, N–H, and C–O stretching bonds. These bands showed that bonds like those found in chitosan, PVA, and pectin were already there. Both experimental groups did not detect any new chemical bonds. This implies that the components underwent physical blending instead of a chemical reaction^63–65^. The observed peak at 1019 cm^−1^ is due to the C–O stretching bond^66^. The C–O stretching bond was observed at 1079 and 1028 cm^−1^, while the C–C stretching bond at 846 cm^−1^ was observed^67^. The bands at 1089 and 1016 cm^−1^ correspond to C–O–C stretching^68^.

The presence of nano-topographic features can promote the healing of wounds and strengthen the integration of implants into bone by facilitating the process of cellular differentiation and proliferation^56^. Osteoblasts typically possess a negative charge that naturally causes them to be repelled by the negatively charged titanium surface. However, this repulsion can be counteracted by other attractive forces in the system, such as proteins with positively charged tips that are located next to the charged titanium implant surface^69^. By augmenting the roughness at the nanoscale scale, the surface charge density and the strength of the electrical field are enhanced, resulting in the generation of bridging attractive forces for protein mediation. The electrostatic attractive contact between matrix proteins and fibronectins enhances the binding of cellular integrins to these molecules, resulting in osteoblast adhesion^70^. This can clarify the importance of analyzing surface topography and roughness characteristics in implant dentistry and how it affects the long-term success of implants.

Natural polysaccharides can play a principal role in biomedical applications since they have a similar structure to an extracellular matrix (ECM). The extracellular matrix facilitates cellular interaction with their environment^71^. The ECM delivers a mechanical encouragement to cellular attachment, signaling cell proliferation, cell coordination, and conservation of cell differentiation. An additional reason for the increased cell viability noted in the coated groups may be due to the better hydrophilic properties of this groups substrates as evidenced by the wettability test in our previous study^27^, which is conducive to improved cell attachment and proliferation^72^.

The potency of chitosan to disrupt bacterial growth with a strong suppressive impact on both Gram-positive and Gram-negative bacteria is well-established^50,73^. The positively charged nature of chitosan permits it to attach to the negatively charged membrane of bacterial cells. This interaction elicits substantial changes to the cell surface through altering the membrane permeability which in turn causes osmotic imbalance and releases intercellular components, eventually triggering cell death^74^. Several studies have confirmed the antibacterial action of chitosan against both gram-positive and gram-negative bacteria^74,75^. These findings contradict a previous study conducted within the field of food packaging which assessed the antibacterial activity of electrospun nanofibers produced from a blend of pectin/chitosan/PVA. The previous study indicated that these nanofibers exhibited significant antibacterial activity against only gram-positive bacteria, with no considerable impact on gram-negative bacteria^63^. A recently conducted study assessed the antibacterial activity of pectin–chitosan-based film containing Ag nanoparticles against gram-negative bacteria (*E. coli*). The authors concluded that this prepared film had good thermal and mechanical properties, simultaneously with excellent antibacterial activity against a gram-negative microorganism^76^. These conclusions emphasize the synergistic effect of pectin and chitosan in inhibiting pathogenic and opportunistic microorganisms.

A recent study evaluated the combined antibacterial effect of chitosan and alginate coatings applied to titanium substrates also against *E. coli* bacteria. The research concluded that this composite hindered bacterial proliferation and the enhanced antibacterial activity could be attributed to the presence of chitosan in the coating^77^. This outcome supports the current study results, although, in the present study, pectin was mixed with chitosan. It is noteworthy that pectin, like alginate, is also a negatively charged natural polysaccharide. The amine group present in chitosan interacts with the negatively charged bacterial cell walls, causing them to deteriorate and resulting in the death of bacterial cells^78^. The enhanced adhesion strength distinguished in PCPC (1:3) can be attributed to the higher ratio of chitosan incorporated, as chitosan has previously been noted for its strong adhesion to titanium surfaces under tensile stress^79^.

In implant dentistry, nano-scale topography that varies from 1 to 100 nm is most widely applied. These nanoscale surfaces with their intricate textures possess higher surface energy, which in turn enhances the surface's wettability by blood and improves cellular attachment. Nano-topographic features can enhance the healing of wounds and bolster osseointegration following implantation by aiding in cellular differentiation and growth^54^. Osteoblasts often carry a negative charge which is naturally repelled by the similarly negatively charged titanium surface unless counteracted by other attractive forces within the system corresponding to proteins with positively charged tips adjacent to a charged titanium implant surface^80^. By increasing the roughness at the nanometer scale through a biocompatible coating layer, there is an increase in both the surface charge density and the intensity of the electrical field to originate bridging attractive forces for protein mediation. This electrostatic attractive interaction leads to the attraction of matrix proteins and fibronectins, which subsequently facilitates the binding of cellular integrins to these molecules and mediates osteoblast adhesion^81^_._

Based on the completed research, it was discovered that titanium-coated substrates with a combination of pectin, chitosan, and PVA, using a 1:3 ratio of pectin–chitosan, showed improved biocompatibility, considerable antibacterial effects, increased surface nano-roughness, and high adhesion strength to the titanium base. This specific blend of pectin and chitosan, known as PCPC, can be considered a highly efficient coating for titanium dental implants. It should undergo additional validation through animal study evaluations that will be examined in the subsequent investigation.

## Conclusions

Research into implant nanocoating faces numerous challenges when employing materials like pectin, chitosan, and PVA through electrospinning/spraying techniques. Key hurdles include achieving consistent coating thickness and uniformity, maintaining stability, ensuring biocompatibility and minimizing cytotoxicity, and optimizing the mechanical properties to withstand physiological conditions. This study highlights the benefits of utilizing pectin–chitosan polyelectrolyte composite (PCPC) to produce an implant coating that is both biocompatible and physically robust while maintaining structural uniformity on commercially pure titanium substrates. The substrates coated with PCPC (1:3) exhibited increased average cell viability and satisfactory antibacterial characteristics after 1, 2, and 3 days. They also presented applicable adhesion strength and notable surface roughness parameters compared to the experimental groups. Based on the extensive in-vitro studies, the PCPC method, which involved using a pectin–chitosan ratio of 1:3 mixed with PVA to enhance the spinnability process, demonstrates promise as a viable coating for commercially pure titanium dental implants. Furthermore, it is necessary to conduct in-vivo studies to validate the findings observed in-vitro within a clinical context. Future animal and clinical studies are poised to validate the safety, efficacy, and biocompatibility of these coatings, aiming to enhance the performance and longevity of dental implants. By refining manufacturing processes to ensure reproducibility and scalability, and by conducting rigorous preclinical assessments to establish optimal coating compositions and application methods, researchers can pave the way for clinical trials. Ultimately, successful implantation in clinical settings could lead to transformative advancements in medical treatments, offering patients improved therapeutic outcomes and reduced risks of implant-related complications.

## Data Availability

All data generated or analyzed during this study are included in this published article.
